# “What being healthy means to me”: A qualitative analysis uncovering the core categories of adolescents’ perception of health

**DOI:** 10.1371/journal.pone.0218727

**Published:** 2019-06-21

**Authors:** Alberto Borraccino, Rebecca Pera, Patrizia Lemma

**Affiliations:** 1 Department of Public Health and Paediatrics, University of Torino, Torino, Italy; 2 Department of Management, University of Torino, Torino, Italy; IRCCS E. Medea, ITALY

## Abstract

**Background:**

Studies exploring adolescents’ perception of health are still scarce in the international literature. Through a qualitative analysis, this study aims to explore the core categories or themes evoked when adolescents describe what it means to be healthy and unhealthy.

**Methods:**

A convenience purposive sample of 34 15-year-old students from three different upper secondary schools took part in a 2-hour group discussion session. During the session, two conceptual projective techniques, the collage creation and the think-aloud technique, were used to elicit perceptions and descriptions of the typical healthy and unhealthy adolescent. Perceptions and descriptions voiced by adolescents were analysed through content analysis, and the key concepts that emerged were grouped so that core categories or themes could be identified.

**Results:**

The analysis revealed five core categories that adolescents used to describe what being healthy or unhealthy meant to them: physical appearance, personal commitment and goals, possessions and space, use of free time, and social belonging.

**Conclusions:**

Instead of those approaches that focuses solely on the avoidance of risk, the identified core categories or themes might be the basics around which health promotion programmes in adolescence should be built. Engaging students in planning for their future and assisting them in mapping out crucial steps to meet their personal goals, including life, academic, and career goals, is a suitable way to address issues that are meaningful to adolescent health.

## Introduction

Research on adolescent health, particularly in the medical preventive field, has traditionally focused on specific behaviours (i.e. smoking, alcohol consumption, and unhealthy diet) and their association with negative health outcomes [1–3]. Only in more recent years have researchers begun to agree on the importance of investigating not only risk factors, but also the role that specific protective factors can have on adolescent health. Protective factors are defined as those ‘*individual or environmental characteristics that reduce the effects of stressful life events*, *increase an individual’s ability to avoid risks or hazards and promote social and emotional competence to thrive in all aspects of life now and in the future*’ [4]. Previous studies have shown that adolescents consider the adoption of high-risk behaviours as a way of expressing distress, or as a consequence of problems originating elsewhere, and problems with school and family relationships seem to play a crucial role [5, 6]. Specifically, evidence has shown that the presence of protective factors in young subjects can effectively decrease the incidence and prevalence of known risk factors [7–9].

The interest in protective factors has intensified, including in the areas of disease and health, as have the efforts to develop and test tools for measuring these factors at both the individual and population levels. Self-administered questionnaires are the most commonly used, particularly in studies of young people. However, most of the existing questionnaires that target adolescent populations have been developed around expert opinions [10]; they seldom take into consideration the ideas and views of adolescents themselves [6]. It is only in more recent years that adolescents were surveyed with the purpose of identifying their subjective definition of health and well-being and their perception of the resources required to pursue them [5, 11, 12]. Authors who explored these issues have used a qualitative approach. Although these studies were developed in different countries and cultural contexts, they have shown that eliciting adolescents’ views on health-related matters that are important to them is a suitable way to obtain a deeper understanding of this issue, and to aid in intervention planning and evaluation [5, 6, 11, 12]. This emic approach, which allows participants to express their ideas and priorities using their own vocabulary, has been shown to not only allow theory building, but also to directly contribute to more accurate programme development [11, 12]. In fact, it has been reported that among the reasons for the limited success of many health promotion initiatives is the fact that policy-makers are prone to mainly focus on experts’ opinions instead of prioritising the views of the target population, and this is particularly frequent when the target population is teens. In contrast, programmes incorporating qualitative approaches that included the adolescents’ voices, are consistent with the population’s views on health, and are more likely to be accepted, leading to overall improvements in the health status of the target population [5, 13].

At present, there is a paucity of studies exploring the perception and meaning that adolescents attribute to their health, particularly among Italian youths. This study aims to explore the core categories or themes evoked when adolescents describe what it means to be healthy and unhealthy.

## Methods

### Ethics approval and consent to participate

The qualitative enquiry study protocol, that included sampling, questionnaire and opt-out procedures, was officially approved by the bioethical committee of the University of Torino under the title ‘Stili di vita e progetto salute’ [Lifestyles and Health Project].

### Setting

The school system in Italy covers 13 years, 10 of which are compulsory. This breaks down into 5 years of primary school (starting at the age of 6 years); 3 years of junior high school; and 5 years of upper secondary school (of which only the first 2 years are compulsory). There are different types of upper secondary schools in Italy, and they offer different educational programmes: 5-year academic programmes offer a path to university, and are available at “gymnasium” or “scientific lyceum” schools; 5-year technical programmes offer a path to employment or university, and are available at technical schools; and 3-year vocational programmes offer a path to employment, and are available at vocational schools. In Italy, there is evidence of a significant relationship between the type of upper secondary school chosen by adolescents and their family’s social position [14].

### Design and participants

To guarantee the desired intra-group heterogeneity, we chose participants from three different upper secondary schools in Turin, a large metropolitan city in the north of Italy: one scientific lyceum school; one technical school; and one vocational school. The three schools were purposively selected from the representative sample of 85 upper secondary schools participating in the 2014 official wave of the Health Behaviour in School-aged Children (HBSC) study in Italy [Health Behaviour in School-aged Children (HBSC) is a cross-national research study conducted in collaboration with the WHO Regional Office for Europe, involving to date 45 European and non–European countries. Two of the authors are members of the National HBSC research group, with scientific responsibility for the Italian country. Further details and study reports can be found in http://www.euro.who.int/en/health-topics/Life-stages/child-and-adolescent-health/health-behaviour-in-school-aged-children-hbsc (last accessed june 2019)].

Written information about the current study’s content was sent to the chosen schools’ directors, and each school designated one teacher to (i) inform students about the qualitative enquiry procedure and (ii) distribute an information sheet and a written opt-out form. The information sheet included basic information about the qualitative enquiry procedure, specifics about the methodology (e.g. number of participants, inclusion criteria, opt-out option), organisation and logistics (e.g. setting), and the content to be discussed. Students were to deliver the information sheet to their parents or guardians, and if parents did not want their student to participate, they signed and sent back the opt-out form. To ensure the adolescents willingness to participate the study, a further oral informed consent was obtained in the classroom before the activities. Designated teachers were then asked to randomly select 12 willing 15-year-olds, both male and female, from among those with parents’ or guardians’ authorisation. In total, 34 adolescents participated—11 from the scientific lyceum school, 11 from the technical school, and 12 from the vocational school (two boys were absent the day of the session). The mean age of participants was 15.3 (SD ± 0.5) years with a male/female proportion of 0.55 (19 male and 15 female participants).

### Instruments

All participants took part in a guided, 2-hour group session in a dedicated room during school hours. In order to help adolescents overcome any reluctance to talk about their feelings, and to gather a collective representation of the issue under study, two conceptual projective techniques (CPTs) were used: the collage creation technique[15] and the think-aloud technique[16]. CPTs are meant to provoke deep thoughts, emotions, metaphors, and unconscious thinking. They have been used in the social sciences to help participants discuss a specific issue using their ideas and their own vocabulary and have proven effective in creating a composite structural representation of the issues under study [15–18].

Collage creation technique: at the beginning of each session, the moderator presented the first activity. Several newspapers and magazines were made available to the group, and the moderator asked each participant to choose images (pictures or part of a text) from these sources that, in their view, represented health and the meaning of being healthy or unhealthy. Participants were free to choose any images they liked, reflecting perceived positive or negative aspects of health [19, 20].

As a second activity, participants were asked to collectively combine the selected materials to create a new, flexible composition of the topic being discussed: one which showed images that embodied their collective idea of a healthy adolescent and another that embodied their collective idea of an unhealthy adolescent (CPT output). Characteristic of this creation of collages was the think-aloud process: participants had to discuss among themselves and verbally agree on which images belonged on which collage and why; they had to describe, in their own words, the specific features of a healthy and an unhealthy adolescent (i.e. lifestyle, activities, hobbies, choices, and personality) [15, 16, 18].

The whole process was unstructured and left to the participants. The moderator’s task was to stimulate the discussion without intruding or judging, avoiding any censorship or any sign of approval to allow for maximum fluidity. The moderator’s aim was to facilitate any and all representations of health and to increase idea-generation among the participants, favouring a process of social sharing of personal views and avoiding any interference. To close each session and to increase data validity, the moderator dedicated some time to summarising and sharing the achieved results, to allow participants to verify the information elicited.

### Analyses

Neither the chosen images nor the final collages were stored or analysed, as they were only used to elicit discussion. Group discussions during the think-aloud process were audio recorded and transcribed for analysis.

Content analysis was used to explore and synthetise participants’ perceptions and descriptions of the whole phenomenon of being healthy or unhealthy. Content analysis is widely used in qualitative research to develop objective inferences about a specific subject of interest through analyses of any type of communication. The process consists of systematic coding of raw messages, in our case textual material. [21]. The entire analysis was performed by two independent researchers through open coding. According to Corbing et al., analyses were started at the end of each session and preliminary emerging concepts were brought into the following sessions to help the moderator in the eliciting process[22]. Qualitative content analyses were then performed on the whole set of data. Identified recurrent concepts that pertained to the same ideas were condensed into key concepts. Selected key concepts were highlighted for similarities and differences, were then grouped and abstracted to produce core categories or themes, inter-observer consensus was guaranteed along the whole process[21–23]. Analyses were performed without any computer-aid tools assistance.

## Results

During the group discussion sessions, participants agreed that the techniques employed allowed them to reflect deeply on their individual experience regarding health and positively enriched the discussion. Analysis of the group discussions yielded five core categories: (1) physical appearance; (2) personal commitment and goals; (3) ownership of possessions and spaces; (4) use of free time; and (5) social belonging (Table 1).

**Table 1:** Core categories and key concepts of the two images that embodied the collective idea of a healthy and an unhealthy adolescent.

Key concepts of being healthy	Core categories	Key concepts of being unhealthy
• A nice person with a good (physical) appearance • In good shape • Average weight	Physical appearance	• Fat, ugly, and dirty • Eats too much and makes bad food choices
• Studies a lot • Participates in after-school courses/activities • Has a focus and goal in life • Has influential friends and luck	Personal commitment and goals	• Lazy and sloppy • Has no extracurricular activities • Is a lonely person
• material resources perceived as important to be part of the peer group • Has a sufficient amount of space within and outside the house	Ownership of possessions and space	• Has familial economic difficulties
• Plays sports • Goes out with friends	Use of free time	• Remains at home, attached to technology • More likely to display deviant, risky behaviours
• Is part of a united, inclusive, well-to-do family • Both parents work • Family is an important resource for everyday life • Has friends with whom he/she shares interests	Social belonging	• Lives in a poor family • Father unemployed or perhaps in early retirement • Main interest is watching television

### Core categories and key concepts

#### Physical appearance

The healthy adolescent was described as a good-looking person with a good (physical) appearance (*‘[…] pretty*, *beautiful*, *but not too much […]’*) and in good shape, i.e. at a healthy weight. He/she *‘[…] pays attention to what he/she eats*: *fruits*, *vegetables*, *white meat’*. The unhealthy adolescent was depicted as *‘[…] fat*, *ugly*, *and dirty […]’* in his/her appearance and behaviours: *‘[…] eats too much and eats poorly*: *candies*, *sweets*, *fried foods and the like’*.

#### Personal commitment and goals

According to the participants, the healthy adolescent displays a strong personal commitment towards school and studies *(‘[…] attends a good grammar [gymnasium or scientific lyceum] school*’, *‘[…] participates in after-school activities’*), whereas the unhealthy adolescent was described as lazy and sloppy *(‘[…] the worse he/she performs at school*, *the more he/she is pleased’)*. He/she *‘is a bench warmer’* and *‘does not feel like working’*.

The healthy adolescent has a focus and a goal in life, which participants translated into having a solid plan for the future, such as a prestigious profession *(‘[…] he/she will surely become a medical doctor or a lawyer*’), along with powerful, influential friends, and luck, whereas the unhealthy adolescent was described as an unsuccessful person or simply unemployed *(‘[…] he/she will do nothing*, *be a bum*’). Additionally, social relationships were not present in participants’ description of the unhealthy adolescent. They were depicted as lonely people who are *‘[…] alone’*.

#### Possessions and spaces

The healthy adolescent was frequently perceived as having possessions or spaces, such as a new phone, a particular game console, or designer clothes. More generally, these concepts were sometimes expressed as the availability of a sufficient amount of space, both within the house (*‘[…] he/she has his/her own bedroom’*) and outside *(‘[…] green space is available in which he/she can walk […] with friends*’). The unhealthy adolescent, on the other hand, was described as having parents who struggle financially *(‘[…] does not have money to pay the mortgage*, *rent*, *or bills; […] he/she can’t even afford to buy textbooks*’).

#### Use of free time

Another important, particularly distinguishing core category was the use of free time: the healthy adolescent was described as someone who plays sports and has fun with friends—and *‘[…] he/she messes up sometimes*, *of course’*. The unhealthy adolescent was depicted as someone who cannot practice any sports because he/she *‘[…] remains locked up at home*, *attached to his/her technology’* and is more likely to adopt deviant behaviours like smoking and drinking, which emerged as recurrent themes: *‘[…] and he/she also smokes his/her joints alone’*.

#### Social belonging

A strong sense of social belonging, both at the family level and the peer level, was described as being important to health. Indeed, the healthy adolescent was described as someone who lives in a united, inclusive, wealthy family in which both parents work (*‘a nice family*, *open and inclusive […] and they also have really big cars’*). Family was constantly depicted as an important resource both for everyday life and for well-being in general: *‘[…] when you have everything and you are not happy with your family*, *ultimately*, *it is as if you have nothing’*.

The unhealthy adolescent was described as belonging to a poor family, with parents who might be unemployed *‘or perhaps forced into early retirement’*, and in which the main family interest is watching television (*‘[…] and when they are lucky*, *they succeed in catching all the scheduled reality TV shows’*).

Participants also said that the healthy adolescent has friends with whom they share interests. This was juxtaposed against the definition of the unhealthy adolescent: *‘[…] if you can’t share what you have*, *it is as if you don’t have anything; […] the whole time by himself*, *alone’*.

## Discussion

In this work, adolescents were considered as experts who could contribute to the collaborative description of the core categories or themes that define a healthy adolescent [5, 11]. In developmental ages, in particular during adolescence, teens gradually acquire a higher level of autonomy in their behaviours, especially those related to their health. To fully understand the meaning attributed to some of these behavioural choices, which can positively or negatively impact health, it should be taken into account that adolescents cannot always take their knowledge and attitudes and express them as reasoned responses to specific life-related questions. Therefore, it is important to engage them in a collaborative disentangling process.

Compared to other research approaches, group discussions have been shown to be an effective method, revealing dimensions of understanding that often remain untapped when using, for example, more conventional one-on-one interviews. Furthermore, group discussions are relaxed situations in which participants can freely discuss and share ideas without feeling pressured to give the ‘right’ answer or respond in a certain fashion, thus enabling the collection of data on group norms, which was also particularly important for the goal of this study [24].

Among adolescents, everyday forms of communication such as anecdotes, jokes, or loose word association may reveal as much, if not more, about personal feelings towards individuals and the surrounding environment [25]. For these reasons, our qualitative enquiry was structured to elicit a detailed description from participants of two juxtaposed adolescents: the ‘healthy adolescent’ who embodies good health, and the ‘unhealthy adolescent’, who embodies a lack of health [26].

Although they were selected from three different types of schools, our participants described similar profiles of healthy and unhealthy adolescents, with several overlapping core categories/themes. The phrasing used by students from the same school was similar, and it was only slightly different across the three schools: students from the scientific lyceum and technical schools demonstrated greater lexical skills than those from vocational schools, but the content of discussions was interestingly similar.

Participants’ depictions of the unhealthy or the healthy adolescent were interestingly far from the simple ‘presence or absence of disease’. Indeed, in their narratives there was no connection with any physical or psychological symptoms, or any medicalised dimension. For example, healthy eating was seen more as a function of physical appearance, as an aesthetic concept, than as a means to improve health [27]. On the same level, well-known risky behaviours—such as smoking, drinking, or using soft drugs—were commonly described as an expression of loneliness[28]. Also, not surprisingly, the idea that friends can act as negative influences was somewhat absent. Previous studies have shown that being alone—specifically, not having friends to rely on—was, in adolescents’ mind, associated with the presence of deviant behaviours, a lack of self-care, and poor time-management skills [5].

Participants described health as a positive and active concept, and the healthy adolescent was described with a particular emphasis on personal commitment, environment, and relationships. As other international studies have recently shown, being healthy was depicted as a functional condition in which an individual can perform activities to achieve his/her goals both in school, as their proximal setting, and more broadly in life [11, 29]. In particular, and in accordance with other studies, having any goal for the future, in contrast to having no goals, was depicted as visible sign of health and was associated with positive well-being[9, 30].

Participants identified social belonging as another key element in the healthy adolescent. The relevance of the family dimension was consistently depicted as an important resource both for everyday life and for well-being in general. Moreover, having positive peer relationships, in which friends can spend time together, support each other, and build each other’s confidence, was also considered a bridge to health. These results have also been confirmed in other research [31] using quantitative approaches. Indeed, positive relationships with parents and peers have been shown to have a protective effect on specific outcomes, for example health complaints[32] and the occurrence of risky behaviours[33]

Another constant mentioned by our participants was how having prestigious and influential friends can be helpful in obtaining a favourable social position, but having a bit of luck has its own importance. Through these concepts, the latter in particular, teens seemed to express the concern that commitment, initiative, and inclination alone were insufficient to help them realise their future goals. Similar to what other studies have shown, having possessions and personal space, both inside and outside the home, also played an important role in evoking an image of health. Environment has been reported to be a strong resource for the development of adolescent health [5]. Furthermore, in recent years, scholars have stated that the materialistic cultural factors that characterise modern times can have an impact perceived well-being [34–36]. For example, the perception that one lacks the resources to acquire the possessions necessary to belong to what one considers their appropriate peer group, can have a negative effect on both physical and psychological health [37].

### Limitations

Our findings, although thought-provoking, should be interpreted while taking into account the weaknesses of study and the study design. A convenience sample was drawn, selecting three specific schools from a representative random pool of upper secondary schools that were eligible for the HBSC study[38]. Moreover, because of the broader qualitative enquiry paradigm, participation was limited to students who were willing to join the group discussion sessions. As such, we could not collect other information or points of view. On a positive note, the overall refusal rate due to parental or student denial was low in all three schools, accounting for less than 5% of students.

The teachers in charge of selecting the participants could have unintentionally introduced selection bias and thereby reduced the heterogeneity of our study sample, for example, by assembling a group of only loquacious students. Homogeneity and heterogeneity are important features to take into account in planning group discussions, and both have to be managed carefully. It is recommended to assemble groups with the homogeneity necessary to allow the highest level of comfort among participants, while still guaranteeing good homogeneity [39]. For these reasons, since participants belonged to schools that were randomly selected as eligible for the HBSC study and were chosen to represent different socio-economic backgrounds, we believe that the potential distortion was somewhat minimised.

Another important caveat could be the total number of group discussions, which was limited to three. A key aspect in qualitative research is the number of groups considered adequate to allow for plausible data saturation. The concept of saturation was introduced by Guest et al. (Guest, 2006) [40] and identifies ''the point in data collection and analysis when new information produces little or no change to the codebook''. Group discussion saturation was empirically assessed in two different studies, which determined that three is the number of focus groups required to reach almost 90% saturation [41, 42].

Lastly, the need to identify two juxtaposed ideas of a(n) healthy and unhealthy individual could also be considered a study limitation, since participants were forced to describe the extremes of a complex continuum. This choice was driven by the intent to disaggregate any intermediate categories in which uncertain participants might have been likely to place their responses[16]. For example, the occurrence of too many intermediate categories could have flattened the evoked images, and had a negative effect on achieving the study aims.

## Conclusion

Consistent with other published findings, our results revealed that adolescents consider a strong connection with peers, the environment, and family; a commitment to education; and a plan for the future to be important protective factors for health [4, 43]. To maximise potential effects, the five core categories or themes we report here might be the core around which health promotion programmes in adolescence should be built, instead of those recurrent approaches that tend to focus on the avoidance of risk. Indeed, school programmes that solely address well-known health risks are likely to be ineffective, since adolescents perceive health as an active functional concept that is closely tied to their environment.

On the content level, according to the National Institute of Clinical Excellence, schools should put more effort into creating effective strategies to foster decision-making processes that facilitate student, family, and community engagement; engaging students in planning for their future; and assisting them in mapping out crucial steps to meet their personal goals, including life, academic, and career goals [4, 44].
